# Nano-Crystal and Microstructure Formation in Fluoride Photo-Thermo-Refractive Glass Using Chirp-Controlled Ultrafast Laser Bessel Beams

**DOI:** 10.3390/nano11061432

**Published:** 2021-05-28

**Authors:** Yunjie Zhang, Xu Wang, Guodong Zhang, Razvan Stoian, Guanghua Cheng

**Affiliations:** 1 School of Science, Xi’an Polytechnic University, Xi’an 710048, China; zyjnwpu@163.com; 2 State Key Laboratory of Transient Optics and Photonics, Xi’an Institute of Optics and Precision Mechanics, Xi’an 710119, China; wangxu@opt.cn; 3 School of Artificial Intelligence, Optics and Electronics (iOPEN), Northwestern Polytechnical University, Xi’an 710072, China; guodongzhang@nwpu.edu.cn; 4 Laboratoire Hubert Curien, UMR 5516 CNRS, Université de Lyon, Université Jean Monnet, 42000 Saint Etienne, France; razvan.stoian@univ-st-etienne.fr

**Keywords:** photosensitive materials, ultrafast nonlinear optics, ultrafast technology, microstructure fabrication

## Abstract

Nano-crystals were formed in the exposed regions of photo-thermo-refractive glass undergoing irradiation with zeroth order chirp-controlled ultrafast laser Bessel beams and subsequent heat treatment. Effects of various writing powers, pulse durations and heat treatment time on the distribution and the size of the nano-crystals were investigated. The results show that nano-crystals’ distribution depended on the laser power density spatial shape, while the size of the nano-crystals is quasi-independent. However, the average diameter of the nano-crystals was affected by the heat treatment time, decreasing from 175 to 105 nm with the time halved. In addition, using crystallographic characterization by X-ray diffraction, the nano-crystal composition in the laser-exposed regions was detected to be sodium fluoride.

## 1. Introduction

As a typical representative of advanced glasses, photo-thermo-refractive (PTR) glass is not only an attractive photo-thermal glass with admirable mechanical and thermal stability, but also has a broad transparency range from 350 to 2700 nm and a high optical damage threshold [1,2,3]. Its refractive index can be designed in magnitude and in sign. Specifically, the permanent refractive index modulation in PTR glass due to light and heat is triggered by light-activated thermal precipitation of bromide, chloride and fluoride crystals, which could be partially tuned by a combination of laser irradiation, electronic excitation and heat treatment [1,4,5]. These properties make PTR glass interesting as a new potential candidate for hologram optical devices [6], advanced laser systems engineering [7,8], optical design and applications in photonics [4]. Since the invention of PTR glass 60 years ago, optical components based on PTR glass have been put into commercial applications in the past decades. However, many issues about the detailed crystallization mechanism still remain to be solved [9,10].

Up to now, systematic investigations have been carried out to unveil the intricate crystal nucleation and crystallization processes of ultraviolet (UV)-exposed PTR glass by experiment and/or simulation of crystallization kinetics. Lumeau et al. [11] analyzed the size of NaF crystals in UV-exposed fluorine-doped PTR glass by observing the broadening of the X-ray diffraction line. Their investigation showed that the size of the crystals was limited to 20 nm due to the exhaustion of Na^+^ and F^−^ within the glass matrix and the raising of the glass viscosity around the crystals. In addition, Lumeau et al. [12] focused on the influences of the cooling steps after heat treatment (HT) on the nucleation, crystallization and the optical features of UV-exposed PTR glass. The results showed that multi-step HT (including some cooling steps) could induce smaller crystals and finer structures than continuously uninterrupted HT. The above studies demonstrate that the conditions of incident laser radiation and HT would directly influence the size and the distribution of nano-crystals in UV-exposed PTR glass. 

Nowadays, with the development of ultrashort pulse laser technology, near-infrared ultrafast laser exposure of PTR glass has gained additional interest with respect to UV exposure due to the capability of confining energy in the volume, triggering nonlinear pumping of free electrons in small spatial domains. Therefore, the ultrafast regime can obtain increasingly complex photonic structures through a simple and effective irradiation process, enabling a range of new applications, including integrated optical systems [13,14], optomechanics [15] and optofluidic devices [16]. Specifically, through electronic excitation, changes of molecular bonding and heat, high-intensity femtosecond (fs) laser pulses can locally change the chemical evolution of the excited transparent material, resulting in elemental separation, changes of valence states and a series of free-electron-intermediated chemical effects [17]. Through the same mechanisms originating in nonlinear photoionization, fs laser pulses determine also a high degree of energy localization in the exposed volume, which creates thermo-mechanical transformations and changes to the local structure, all of which are localized in space. Consequently, different from linear UV exposure, the nonlinear photoionization and local structural changes induced by fs lasers can directly modulate index locally and provide free electrons to enable local chemistry, and may create structural dislocations as nucleation sites inside PTR glasses at arbitrary locations in the bulk. It is worth noting that, according to the focusing conditions, the propagation of the fs laser pulses can generate intense and narrow filaments in transparent isotropic media [18,19,20]. This propagation balancing diffraction indicates also alternative concepts of irradiation, with an intrinsic non-diffractive nature, one example being Bessel beams [21]. The focused ultrafast laser Bessel beams can uniformly maintain a relatively long distance and have an intense central core [22]. These beams are formed by the coherent conical intersection of waveforms generated by interference patterns resulting in a non-diffractive appearance. In comparison with Gaussian filaments, Bessel standing light filaments have a higher aspect ratio and a higher degree of nonlinear stability [20]. Therefore, such a type of long filament structure opens a way for several applications including photolithography [23], multiphoton microscopy, laser welding [24] and micromachining [25]. For example, transmission volume gratings were fabricated in PTR glass using zero-order fs laser Bessel beams in our previous work [26]. Additionally, the effects of the gratings thickness, writing laser power and thermal treatment on the diffraction efficiency were investigated. However, the distribution of nanocrystals has an important influence on the physical properties of the exposure area in PTR glass, the relationship between the power density distribution of the fs laser and the distribution of nanocrystals requires further study, and the effect of heat treatment time on the size of nanocrystals need to be revealed. 

In this work, the interaction of fs laser pulses focused as Bessel beams into a PTR glass was investigated. The impact of ultrafast laser conditions and HT on the crystallization process inside the PTR glass within the laser exposure areas was studied. The influences of writing power, pulse durations and HT duration on the size and distribution of nano-crystals are discussed in detail. 

## 2. Materials and Methods

### 2.1. Irradiation Process

**Figure 1:**
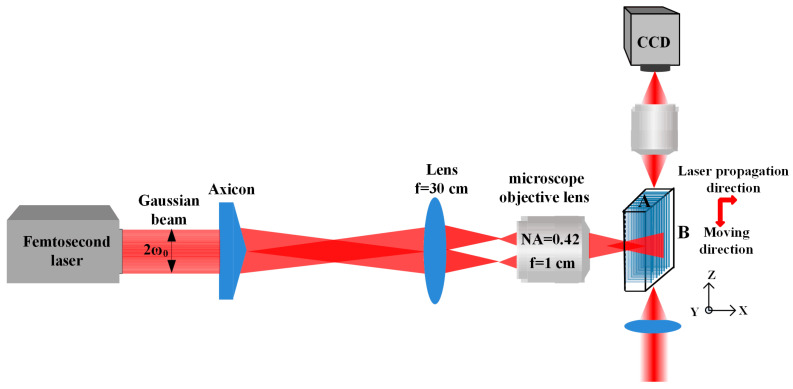
Schematic diagram of the experimental setup for writing parallel tracks.

An amplified Ti: Sapphire laser system working at 800 nm with a maximum power of 600 mW, a repetition frequency of 50 kHz and an adjustable pulse duration from 120 fs to 7 ps was employed as an irradiation source. The incoming fs laser Gaussian beams were transformed into Bessel beams through an axicon lens and then were further demagnified and imaged using a 4f focal system as shown in Figure 1. The 4f focal system was composed from a convex lens of 30 cm focal length and a microscope objective lens (M-Plan, 20X, 0.42NA, Mitutoyo, Tokyo, Japan) with 1 cm focal length. Therefore, the Bessel beam generated was demagnified (demagnification factors of 30) and focused on the inside of the PTR glass. The base angle and material refractive index of the axicon were 0.5° and 1.45, respectively. The radius w_0_ of the incident Gaussian beam was 3800 µm, and the demagnification factors was 30. By calculation, the half-cone angle, diffraction-free propagation distance and radius of the main lobe of the Bessel beam in the PTR were 4.5°, 1602 µm and 2.6 µm, respectively. The intensity distribution of the Bessel beam was relatively uniform in its propagation direction. Further details of the schematic drawing of the zeroth order Bessel beams were described elsewhere [26]. 

### 2.2. Sample Preparation

PTR glass with a composition of 73SiO_2_–11Na_2_O–7(ZnO+Al_2_O_3_)–3(BaO+La_2_O_3_)–5NaF–1KBr (mol%) doped with 0.02SnO_2_–0.08Sb_2_O_3_–0.01AgNO_3_–0.02CeO_2_ (mol%) was used in the work. The glass was cut in parallelepiped forms of 10 mm × 5 mm × 2 mm and then polished. The movement of the glass relative to the laser beam was manipulated by three-dimensional X-Y-Z motorized translation stage. By translating the sample perpendicular to the writing beam, a sequence of parallel Bessel filaments with an interval of 5 µm was written 100 µm below the front surface. The writing velocity was fixed at 200 µm/s. After laser exposure, the PTR glass went through HT for atomic silver nucleation for 5 h at 450–490 °C and fluoride crystallization for 3 h at 520–550 °C. 

### 2.3. Characterization

Optical transmission microscopy (OTM) and positive phase contrast microscopy (PCM) from Olympus (BX51, Tokyo, Japan), were principally used to unveil the morphological characteristics. The affected area was then exposed to chemical etching using hydrofluoric acid. Similar to the fused silica (a-SiO_2_) [27], the rate of hydrofluoric acid chemical etching of nano-crystals in PTR glass was much higher than that of the PTR glass matrix. Therefore, in order to observe the traces of crystals, the samples were polished to the surface and acid treated. Additionally, the corrosion traces of nanocrystals could reflect the distribution and size of nanocrystals. For convenience, the measured nanocrystals were actually the corrosion traces of nanocrystals in this paper. The microstructures of nanocrystals in laser exposure regions were examined using scanning electron microscopy (SEM, EVO-18, Zeiss, Jena, Germany). Crystallographic characterization in laser exposure regions was realized by using an X-ray diffractometer (XRD, Max-2400, Rigaku, Tokyo, Japan) using Cu-Kα radiation.

## 3. Results

### 3.1. Microstructure of PTR Glass under Various Writing Power

**Figure 2:**
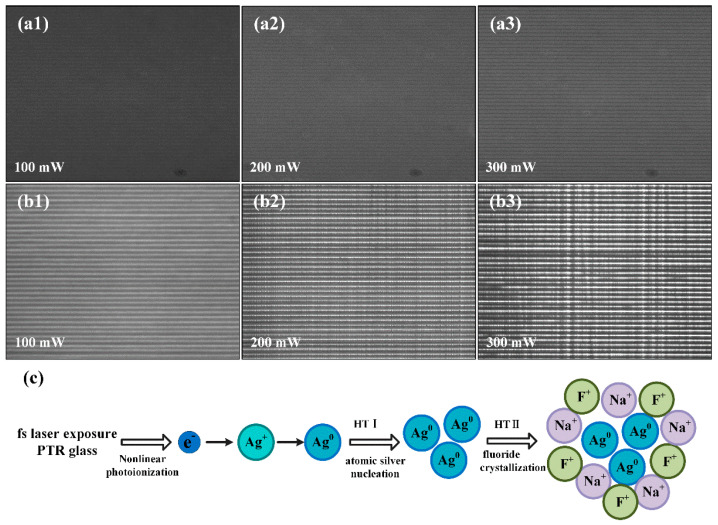
PCM images of parallel tracks before HT (**a**) and after HT (**b**) with different writing powers of 100, 200 and 300 mW. The index change is positive following laser action and turns negative following heat action. (**c**) Schematic for the nonlinear photo-thermo-induced crystallization mechanism in PTR glass.

Figure 2a,b displays the PCM images of parallel tracks with different writing powers of 100, 200 and 300 mW before and after heat treatment (HT), respectively. As presented in Figure 2a, when the fs laser Bessel beams irradiated to PTR glass, the refractive index increased in the focal regions. This could be due to the interaction between the PTR glass and fs laser Bessel beams, which results in a series of complex changes in the PTR glass, including structural transitions and nonlinear photoionization. These interactions could cause local structural modification and densification of the glass for fs laser Bessel beams following bond-breaking, but the specific process is difficult to assess for complex multicomponent glass [28]. By comparing Figure 2a,b, it was obtained that the refractive index changed from positive modulation to negative modulation after HT. The main reason for this phenomenon can be obtained from Figure 2c, which is a schematic describing the evolution of the sodium fluoride nanocrystals after fs laser irradiation and HT. First, the fs laser pulse can trigger a large amount of multiphoton ionization and produce free electrons [29]. The photoinduced electrons are mainly grabbed by adjacent silver ions to form silver atoms. During a two-step HT, silver atoms agglomerate to silver molecular clusters, and then the nano-sized sodium fluoride crystals accelerate growth on it. Since the refractive index of the sodium fluoride crystals is smaller than the refractive index of the glass matrix, nanocrystals are regarded as the cause of the greater but negative refractive index difference after HT. 

**Figure 3:**
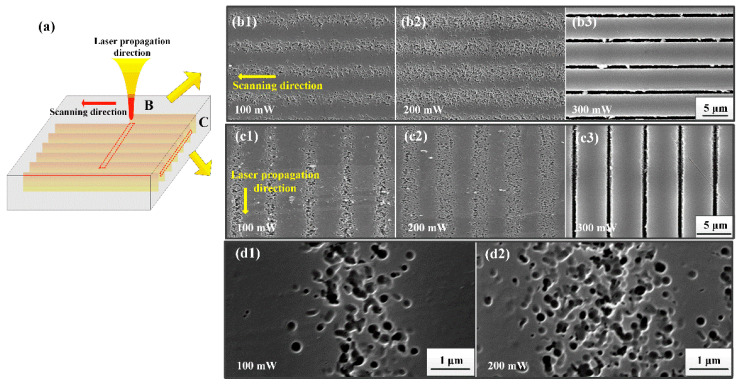
Schematic diagram of Bessel beam propagation inside PTR glass (**a**). SEM images of nano-crystal formation along scanning direction (**b**) and along propagation direction (**c**) with different writing powers. The analysis was performed after HF etching. Note the change in the trace morphologies between low and high power regimes. Magnified views (**d**) of laser scanning direction with writing powers of 100 and 200 mW.

In order to inspect the size and distribution of nano-crystals inside the laser exposure area, parallel tracks with different writing power were perpendicularly (transverse) written in PTR glass by the zeroth order Bessel beams. As indicated before, the index diffractive in those tracks increased after fs laser exposure and then decreased after heat treatment. Figure 3a displays the schematic diagram of transverse written tracks inside the PTR glass. The SEM images of transverse and longitudinal cross-sectional morphology of the tracks (yellow dotted areas) under different writing powers of 100, 200 and 300 mW and at a pulse duration of 200 fs are shown in Figure 3b,c, respectively. The magnified morphologys of nano-crystals along the laser propagation direction are displayed in Figure 3d. A significant difference of morphology can be distinguished between exposed and unexposed regions. Furthermore, the traces of nano-crystals can be clearly observed in exposed regions under low writing power. It is obvious that the concentration of nano-crystals depended on the writing power, but the size of the nano-crystals was found to be quasi power-independent. By statistical calculation, the mean size of crystals was 175 ± 50 nm. As the writing power was brought up to 300 mW, the tracks gathered in a narrow range and formed grooves after acid treatment. 

### 3.2. Nanostructure of PTR Glass under Various Pulse Durations

**Figure 4:**
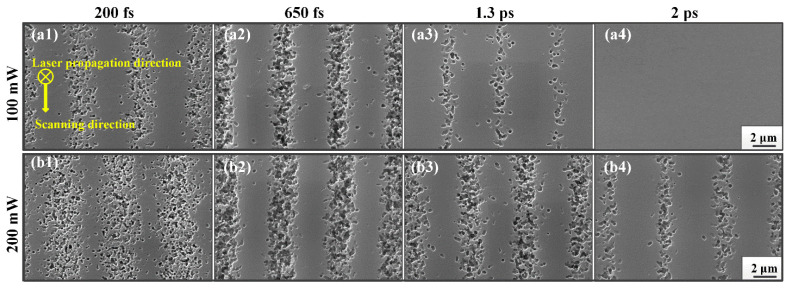
SEM images of nanocrystal traces in PTR glass under different writing powers and different pulse durations: (**a1**) 100 mW and 200 fs, (**a2**) 100 mW and 650 fs, (**a3**) 100 mW and 1.3 ps, (**a4**) 100 mW and 2 ps, (**b1**) 200 mW and 200 fs, (**b2**) 200 mW and 650 fs, (**b3**) 200 mW and 1.3 ps, (**b4**) 200 mW and 2 ps.

Figure 4 presents the SEM images of nanocrystals obtained for various laser conditions in the micron scale traces, allowing to deduce the relationship between the nano-crystal distribution and laser pulse durations in PTR glass at 100 and 200 mw writing power, respectively. With the increase of laser pulse durations, the nano-crystal distribution went through three stages of changes inside the exposed regions. In the first stage, laser pulse duration was relatively short. This duration could trigger nonlinear absorption processes on the laser front, increase ionization and defocus light, which would weaken the laser peak intensity and reduce the density of nano-crystals, as shown in Figure 4a1,b1. There is always an optimum for energy concentration, where the incoming light compensates for defocusing, and the exposure to 650 fs pulses seems to have achieved a higher energy concentration. As the pulse duration slightly increased, the generation of carriers was less swift and the plasma defocusing became less pregnant during the pulse, which allowed concentrating axial energy and accumulating excitation density. Therefore, the nano-crystals distribution became narrower and more compacted, as demonstrated in Figure 4a2,b2. Then, if one continues to increase pulse duration, the intensity drops and the efficiency of excitation drops. In this third stage, the axial energy is not enough to produce a strong nonlinear ionization phenomenon and release free electrons to silver ions when the pulse width increases beyond a certain value. Thus, the phenomenon of nucleation and crystal growth cannot be produced in the laser exposure regions. Based on the above analysis, as the pulse duration increased, the exposure regions of the laser gradually became narrow and compact and then became sparse until they disappeared, as shown in Figure 4a3,a4,b3,b4.

### 3.3. Microstructure with Various HT Times

**Figure 5:**
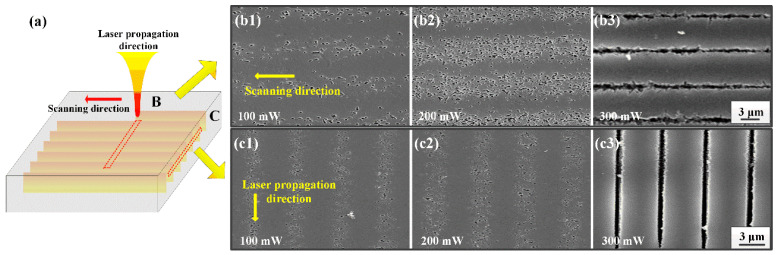
Schematic diagram of Bessel beam propagation inside PTR glass. (**a**) SEM images of nano-crystal formation along scanning direction (**b**) and along propagation direction (**c**) with different writing powers after HT time halved.

Figure 5 displays the distribution of nano-crystal traces within the microtraces produced by fs laser Bessel beam (200 fs) exposure after HT time halved. Similar to Figure 3, as the writing power increased, the tracks containing nano-crystals began to become wider and finally formed clusters at the laser propagation and scanning directions. The observation of the micron-sized structure of the traces containing nano-sized crystals shows that the size of nano-crystals is unrelated to the writing power. However, the density and distribution of nano-crystals were directly affected by the beam shape and the power density distribution of the fs laser Bessel beam. With the increasing incident power from 100 to 200 mW, the laser energy obviously expanded in the transverse direction, and thus the distribution of nano-crystals became dense as the interaction region became broad due to higher laser power. Additionally, the laser power density was gathered in a narrow range with a high writing power of 300 mW. In this process, it is worth noting that spontaneous crystallization was induced in the unexposed regions during HT, but the nucleation-induced photo-crystallization (in the exposed regions) occurred faster than the pure thermally induced spontaneous crystallization (in unexposed regions) [30]. 

**Figure 6:**
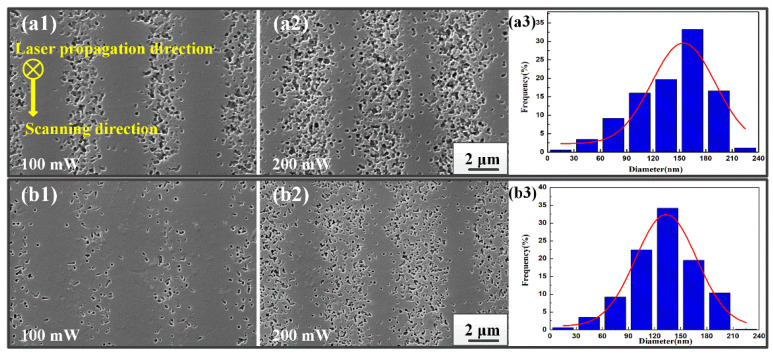
SEM images of nano-crystal formation in PTR glass under different HT times: (**a**) normal time; (**b**) half normal time.

The evolution of the nanocrystal traces varying with the HT time is illustrated in Figure 6. Figure 6a presents the SEM images of crystal distribution under different powers (100 and 200 mW) with a normal time of HT. Figure 6b reveals the change of crystal growth inside PTR glass with the time of HT halved. A very fine structure can be observed in the glass matrix around these crystals. However, when the HT time was only a quarter of the normal HT time, it is difficult to observe the traces of nanocrystals by SEM, which could be attributed to the small size achieved in such a short growth time. Figure 6a3,b3 plot the size histogram of nanocrystals distribution under normal HT time and half HT time, respectively. The nanocrystals kept a nearly spherical shape and distributed in the fs laser exposure area. It is noted that the average diameter of the nano-crystals decreased from 175 to 105 nm with the time of HT halved. Thus, the size of nano-crystals is closely related to the HT time, and different sizes of nano-crystals can be obtained by controlling the HT time. This provides valuable reference data for studying the effect of HT on the nanometer scaled structures formed in PTR glass by light exposure. The detailed relation between crystallization and HT will be studied by changing the HT temperature, the time of HT and multi-step HT in our future work.

### 3.4. Crystallographic Characterization

**Figure 7:**
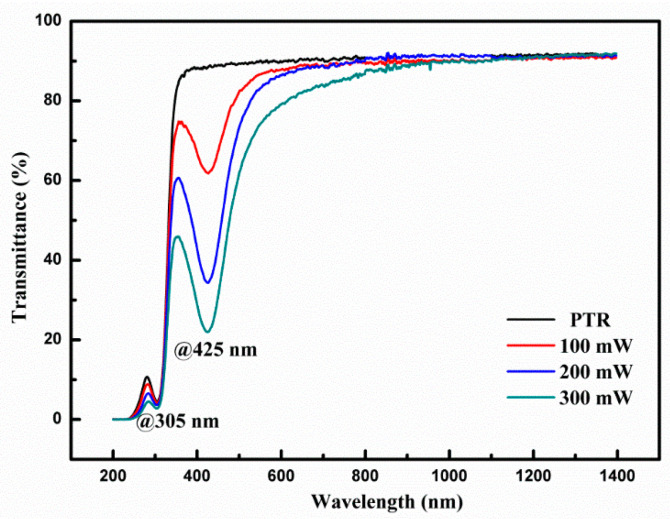
Transmission spectra of PTR glass showing intrinsic absorption at 305 nm by Ce^3+^ dopants and the additional absorption at 425 nm due to silver clusters.

Figure 7 displays the transmission curves of pristine PTR glass and modified PTR glasses after fs laser exposure and HT in various conditions. The black line represents the original transmission curve of the PTR glass matrix in the UV-IR region. It can be seen that there is an absorption peak at 305 nm because of the absorption of Ce^3+^ to ultraviolet light. From the red, blue and green lines it can be seen that the PTR glasses after fs laser exposure and HT showed an absorption peak at 425 nm due to the dispersed colloidal silver molecular clusters in the PTR glass. It is worth noting that there was still a Ce^3+^ absorption peak at 305 nm after fs laser exposure. This phenomenon indicates that the fs laser can directly induce nonlinear photoionization and release a large number of free electrons in PTR glass. Therefore, silver ions can trap free electrons to form silver atoms. Within the nucleation temperature, silver atoms accumulate to form silver molecular clusters and these silver molecular clusters can serve as nucleation centers. Then, nanocrystals can grow around the silver nucleation within the crystallization temperature. More detailed and specific explanations of the transmission spectra have been studied in our previous work [31]. 

**Figure 8:**
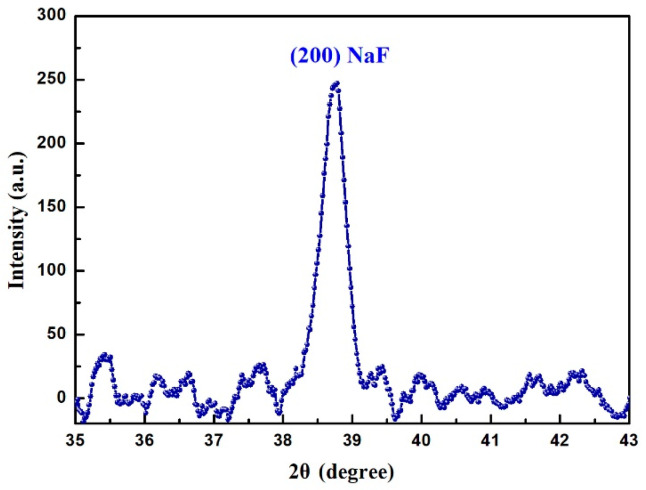
XRD pattern of the PTR glass after fs laser exposure and HT showing the onset of crystallization.

The sample was then pounded into powder and the XRD analysis result is displayed in Figure 8. The purpose of using powdered samples here is to obtain samples with similar properties and avoid the problem of the NaF depletion layer near the surface [11], which causes greater dispersion in the results than in powdered samples. It is well known that before laser exposure and HT of PTR glass, fluorine, sodium and all other ions are dissolved in the matrix and the material is completely glassy. In other words, the original untreated PTR glass exists in an amorphous state. However, the PTR glass after fs laser irradiation and heat treatment has a distinct crystal structure. The diffraction peak of the sample at 38.76° corresponded to the (200) reflections from the crystalline structure of NaF. The results indicate that this type of treatment of PTR glass resulted in the formation of sodium fluoride nano-crystals. The main reason for this phenomenon could be explained as follows. A focused fs laser could trigger a nonlinear ionization phenomenon and release a large number of free electrons in laser-exposed regions. These free electrons can be captured by silver ions and then silver ions convert to silver atoms. On the other hand, the high peak power fs laser can also produce a large number of point defects in the laser focused regions. After laser exposure, the first step of HT leads to agglomeration of silver atoms and acts as a nucleation center. After the second step of HT, nano-crystals would grow around silver nucleation centers and point defects in laser-exposed regions. According to the experimental results, it is concluded that sodium fluoride nanocrystals appeared in aggregation after laser exposure and HT. It is worth noting that the diffraction peak intensity is weak in Figure 8. The main reason for this phenomenon is that the ground powder contains a large amount of originally amorphous PTR glass, and the diffraction peaks can only come from the small irradiated portions of the sample. In order to further increase the intensity and accuracy of the diffraction peaks, we could write a series of parallel tracks without an interval period in PTR glass and precisely polish the unexposed area in our future work.

## 4. Conclusions

In the present work, nano-crystals were observed inside PTR glass after fs laser Bessel beams exposure and subsequent HT. The size and distribution of nano-crystals were studied under various laser writing powers, pulse durations and HT conditions. The microstructure of nano-crystals revealed that the density and distribution of nano-crystals were directly affected by the beam shape and the power density distribution. In addition, the relationship between crystal distribution and pulse durations was studied. The experimental results indicate that the regions of the nano-crystals became narrow and the nano-crystal distribution became first dense and then sparse with the increase of the pulse duration. Additionally, the average diameter of nano-crystals was dependent on HT time, decreasing from 175 to 105 nm with the time of HT halved. These results demonstrate that the distribution and size of crystals could be manipulated by the writing laser conditions and HT, respectively. In addition, nano-crystals in laser-exposed regions were detected to be sodium fluoride by XRD. 

## Data Availability

Data is contained within the article.
